# A Review on Strain Study of Cuprate Superconductors

**DOI:** 10.3390/nano12193340

**Published:** 2022-09-25

**Authors:** Jian Zhang, Haiyan Wu, Guangzhen Zhao, Lu Han, Jun Zhang

**Affiliations:** 1 Institute of Environmental Research at Greater Bay Area, Key Laboratory for Water Quality and Conservation of the Pearl River Delta, Ministry of Education, Guangzhou University, Guangzhou 510006, China; 2 Key Laboratory of Spin Electron and Nanomaterials of Anhui Higher Education Institutes, Suzhou University, Suzhou 234000, China; 3 School of Pharmacy, Dali University, Dali 671000, China

**Keywords:** cuprate superconductors, lattice mismatch, strain, heterointerface

## Abstract

Cuprate superconductors have attracted extensive attention due to their broad promising application prospects. Among the factors affecting superconductivity, the effect of strain cannot be ignored, which can significantly enhance or degrade superconductivity. In this review, we discuss and summarize the methods of applying strain to cuprate superconductors, strain measurement techniques, and the influence of strain on superconductivity. Among them, we pay special attention to the study of strain in high–temperature superconducting (HTS) films and coating. We expect this review can guide further research in the field of cuprate superconductors.

## 1. Introduction

Superconductors have two defining properties: (1) vanishing of electrical resistivity below a critical transition temperature (*T*_c_) and (2) expulsion of magnetic flux below a critical field (*H*_c_). The former was discovered by Kamerlingh–Onnes in 1911, and the latter by Meissner and Ochsenfeld in 1933. YBa_2_Cu_3_O_7−δ_ (YBCO) was the first discovered superconductor that has *T_c_* above the liquid N_2_ temperature (77 K) [1]. YBCO’s discovery fueled a great deal of research activity in superconductivity. Initially, most research focused on developing superconductors with a higher *T*_c_ and critical current density (*J*_c_). Subsequently, other superconductors such as HgBa_2_Ca_2_Cu_3_O_8+δ_ [2] (HgBCCO), Tl_2_Ba_2_Ca_2_Cu_3_O_10+δ_ [3] (TlBCCO), and Bi_2_Sr_2_Ca_2_Cu_3_O_10+__δ_ [4] (BSCCO) have also been discovered that had *T_c_*s above 77 K. Since the common property of all these materials was the layered crystal structure that contained one or more CuO_2_ planes, they were called “cuprates”, which are structurally, chemically, and/or electronically inhomogeneous at the nanoscale.

It is believed that high–temperature superconductors (HTSs) are most suitable for fabricating a lot of potential applications, including motors, magnetic and flywheel energy storage systems, wind generators, magnetic resonance image (MRI), fault current limiters, filters, superconducting quantum interference devices (SQUIDs), and so on [5,6,7]. The field of applied, technological superconductivity is now moving beyond these preliminary demonstrators to the industrial development of commercially viable machines and devices. Strain is a key parameter for understanding many physical phenomena at the nanoscale. The mechanical and electronic properties of a material are directly related to the strain in the material, and the response of a material to an applied strain is fundamental to the engineering of mechanical or electronic properties. Superconducting performance is strongly influenced by strain, which is an unexpected phenomenon [8]. Researchers have spent a considerable amount of time investigating the strain to gain a deeper understanding of its relationship with the film’s superconducting performance [9]. For La_1.9_Sr_0.1_CuO_4_ superconducting films deposited on LaSrAlO_4_ substrates, the compressive epitaxial strain caused by lattice mismatch doubled *T*_c_ [10]. Researchers have observed that anomalous strain fields surrounding misfit dislocations can change the functional properties of oxide heterointerface structures [11,12]. A decrease in *J*_c_ with increasing axial tension or compression in self-field has also been observed in YBCO [13,14,15]. It has been shown in theoretical studies that modulating the heterointerface strain can either improve [16,17,18] or demolish the superconductivity [19]. Therefore, the widespread application of HTSs requires an in-depth understanding of the relationship between composition, functionality, and microstructure.

Our review aims to provide an overview of recent advances in strain studies related to cuprate superconductors. In the first section, we summarize the various methods currently available for applying strain to superconducting materials; the second section describes the diverse characterization techniques for measuring strain; the third section presents a comprehensive description of the influence of strain on superconducting properties. Finally, the challenges and development prospects of current theoretical studies on the impact of strain on superconductivity are discussed.

## 2. Strain Application Methods

**Figure 1:**
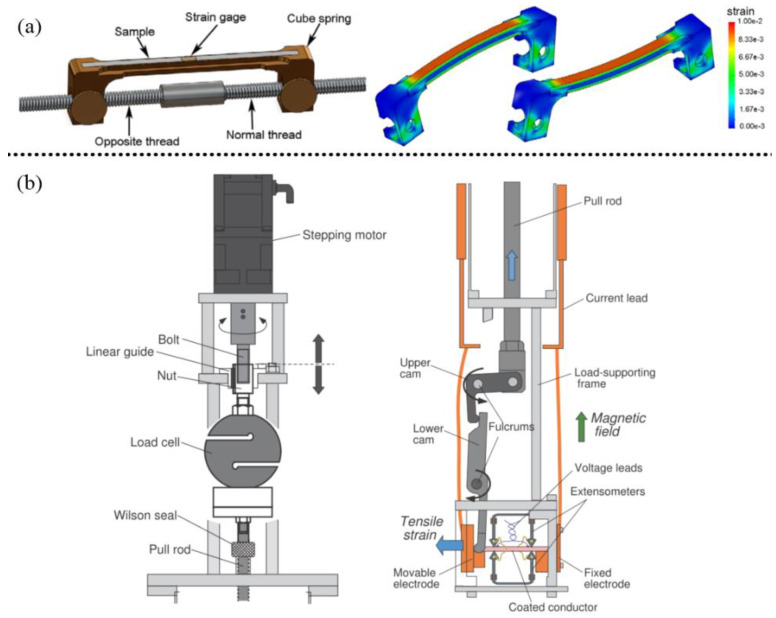
(**a**,**b**) Different methods of applying strain. Reprinted with permission from Refs. [14,21]. Copyright 2007, AIP Publishing; copyright 2010, IOP Publishing Ltd.

**Figure 2:**
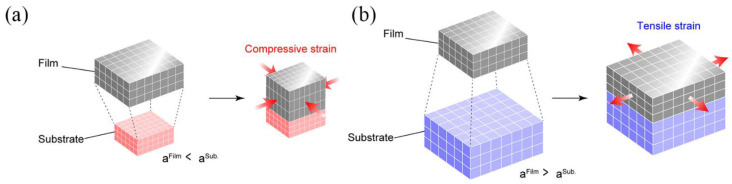
(**a**) *a*^Film^ < *a*^Sub^; compressive strain. (**b**) *a*^Film^ > *a*^Sub^; tensile strain. Adapted with permission from Ref. [22]. Copyright 2020, American Chemical Society.

For superconducting tapes or wires, the strain can be applied by stretching with a motor, as shown in Figure 1. The sample is stretched by mechanical motion to obtain the superconducting performance under strain. However, this approach cannot be applied to HTS films. HTS films are deposited on single crystal substrates that are brittle and will break in the stretched state, failing the experiment. Fortunately, owing to the lattice parameter difference between substrates and films, it is an effective way to apply lattice mismatch to introduce strain into HTS films and study the influence of strain on the superconductivity of HTS films, as shown in Figure 2. Biaxial pressures can be imposed by strain relaxation mechanisms caused by lattice mismatch between films and substrates, which either increases or reduces *T*_c_ [20]. In addition, in artificial flux pinning centers (APCs) studies of HTSs, strain is also generated between the secondary phase and HTS matrix due to lattice mismatch. This is also a very important research direction in the vortex pinning study of HTSs.

## 3. Strain Measurement Techniques

At present, the strain measurement techniques for HTS are relatively mature, mainly including X-ray diffraction (XRD)/ Transmission electron microscope (TEM)/Raman/Neutron Diffraction/Electron backscatter diffraction (EBSD), etc. In this section, we briefly summarize the relevant applications in strain studies of superconductors.

### 3.1. XRD

Jingfeng Yu et al. [23] investigated YBCO films prepared on (LaAlO_3_)_0.3_-(Sr_2_AlTaO_6_)_0.7_ (LSAT), SrTiO_3_, and LaAlO_3_ substrates, and the corresponding influence on superconductivity. Internal strain and residual stress were measured by an extended small-angle sin^2^*ψ* method and the Williamson-Hall plot method, respectively. It was confirmed that YBCO/LaAlO_3_ samples exhibited compressive stress, but YBCO/LSAT and YBCO/SrTiO_3_ samples exhibited tensile stress. The results of their study demonstrated that *T*_c_ had the same variation trend as strain. They suggested that controlling adequate microscopic internal strain and macroscopic residual stress are critical to tailoring microstructures and superconductivity.

Ziliang Li et al. [24] studied the superconducting properties and microstructure of chemical solution deposited (CSD) YBCO films by XRD measurements and magnetic susceptibility. With film thickness decreasing down to 5 nm, the effect of Y_2_Ba_4_Cu_8_O_16_ intergrowth in films was revealed. Ultrathin films offer a unique opportunity to investigate the superconducting properties of highly concentrated nanoscale defects due to the elastic energy related to the misfit strain. Their results demonstrated that superconducting volume decreased strongly correlated with intergrowth volume fraction increasing. Furthermore, they proved that these intergrowths were non-superconducting nanoscale regions that suppressed Cooper pair formation, supporting their role as vortex pining in YBCO films and coated conductors.

### 3.2. Neutron Diffraction

Kozo Osamura et al. [25] precisely investigated the strain of YBCO coated conductors and their corresponding effect on critical current (*I*_c_). The internal strain of the YBCO layer was characterized at 77 K by the neutron diffraction technique. The force-free strain (*A*_ff_) is defined as the point in the YBCO layer at which the internal uniaxial stress becomes zero, where the initial compressive strain decreases during tensile loading and changes to a tensile component. The *A*_ff_ was assessed to be about 0.19 ~ 0.21% at 77 K. A uniaxial tensile load was used to measure *I*_c_ at 77 K. *I*_c_ maximum was observed at 0.035% for the strain dependence. As a result, YBCO-coated conductors’ strain at the maximum *I*_c_ does not correlate with their *A*_ff_.

### 3.3. Raman Spectroscopy

Currently, Raman spectroscopy has been used to probe phonons and other types of low-energy excitations in HTS, which can be applied to analyze internal strains and evaluate possible oxygen loss. For example, Sofia Favre et al. [26] deposited YBCO films by Pulsed Laser Deposition (PLD) and studied the connection between superconductivity and strain. They also studied oxygen loss effects and strain by Raman spectroscopy. The films exhibited an in-plane residual compressive strain, whose degree was determined by film thickness and growth conditions, which influenced the superconductivity. An explanation for the nearly linear depression of *T*_c_ with *c*-axis expansion has been proposed using mutual Coulomb screening between consecutive CuO_2_ planes in the structure.

### 3.4. EBSD

Anjela Koblischka-Veneva et al. [27] performed a meticulous analysis of transmission Kikuchi diffraction (TKD) and electron backscatter diffraction (EBSD) data acquired on various YBCO samples doped with Y_2_BaCuO_5_ (Y-211) nanoparticles. The crystallographic parameter difference between Y-211 and YBCO will introduce residual strain around such embedded Y-211 nanoparticles within the YBCO matrix down to the nanometer scale. Researchers found that the strain around clusters and Y-211 nanoparticles was so large that subgrains formed in YBCO matrix. In addition, they discussed the effect of strain distribution on vortex pinning because stress or strain can provide another source of vortex pinning.

### 3.5. TEM

**Figure 3:**
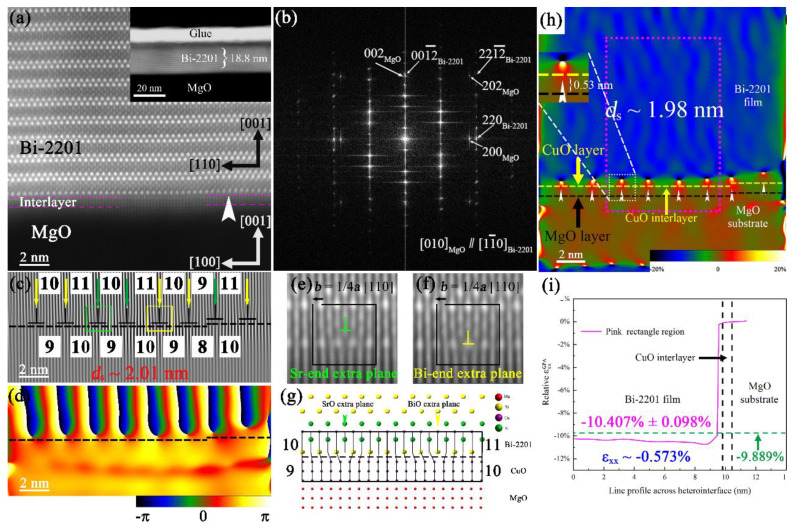
(**a**) HAADF image of the MgO/Bi-2201 heterointerface. (**b**) FFT image of (**a**). (**c**) (200)MgO Bragg and (**d**) geometric phase image of (**a**). Enlarged areas of the green (**e**) and yellow (**f**) squares in (**c**). (**g**) Schematic diagram of 9 or 10 (200)CuO lattices match with 10 or 11 (220)Bi-2201 lattices. (**h**) The ε_xx_ map of (**a**). (**i**) ε_xx_ along the length of the pink rectangle area in (**h**). Reprinted with permission from Ref. [29]. Copyright 2021, Elsevier.

Currently, TEM has been widely applied in the field of microscale strain measurement, and the strain of various materials has been well studied. In previous research, the authors analyzed the heterointerface structure and strain of Bi-based HTS films deposited on SrTiO_3_ [28] and MgO [29] single crystal substrates and demonstrated that the combined effect of lattice mismatch and thermal expansion mismatch caused HTS films to behave differently from the expected strain state (expected tensile strain, but actually compressive strain), as shown in Figure 3.

In general, four main TEM techniques have been created to measure strain, including high-resolution (S)TEM [28,29,30,31,32,33], nanobeam electron diffraction (NBED) [34,35], convergent-beam electron diffraction (CBED) [36] and dark-field electron holography (DFEH) [37]. Strain measurements depend on their spatial resolution for precision and accuracy. As spatial resolution increases, the technique becomes less accurate. The corresponding information is shown in Table 1. For a specific discussion, see previous reviews, e.g., Martin J. Hÿtch et al. [38], David Cooper et al. [39], and A. Béché et al. [40].

## 4. Effect of Strain on Superconducting Performance

Strain has been proved to be an efficient method of modifying superconductivity [41,42,43]. Experimentally, using compressive epitaxial strain, J.-P. Locquet et al. [10] doubled *T*_c_ in La_1.9_Sr_0.1_CuO_4_ films, which has led to a surge in research to improve the performance of superconducting films by applying strain. In this section, we review the effect of strain on La_2−x_Sr_x_CuO_4+δ_, Bi_2_Sr_2_Ca_n−1_Cu_n_O_2n+4+δ_, *RE*Ba_2_Cu_3_O_7−δ_, and other superconducting systems. Moreover, we also focus on the effect of strain on the secondary phase vortex pinning, and the application of buffer layers to release strain.

### 4.1. Effect of Strain on La_2−x_Sr_x_CuO_4+δ_ System

In contrast to other cuprate superconductors, the La_2−x_Sr_x_CuO_4+δ_ (LSCO) system has a lower variation of excess oxygen content, making strain less likely to cause oxygen loss. Therefore, it is a perfect system to study the strain required to increase *T*_c_ [44,45,46,47].

I. Bozovic et al. [48] extended the work of J.-P. Locquet et al. [10] and showed that oxygen intake in such films could cause startling effects such as a crossover from semiconductor to metallic states and colossal lattice expansion. *T*_c_ was improved to 40 K in La_2_CuO_4_ films deposited on SrTiO_3_ substrates without any Sr doping and under tensile strain. On LaSrAlO_4_ (LSAO) substrates, *T*_c_ was improved to 51.5 K, which is an excellent result for the LSCO system.

Eun-Mi Choi et al. [49] deposited self-assembled vertically aligned La_2_CuO_4+δ_+LaCuO_3_ nanocomposite films by PLD and observed weak signatures of superconductivity at ~ 120 K (maximum *T*_c_ ~ 40 K in bulk La_2_CuO_4+δ_) by DC magnetic susceptibility measurements. The 120 K superconductivity occurred only when films contained both *a*- and *c*-axis oriented La_2_CuO_4+δ_ grains. By analyzing lattice parameters close to grain boundaries, it was demonstrated that the expansion of the La perovskite block allowed grains with differently oriented orientations to interact. It is consistent with an interfacial region with a higher *T*_c_. By strain engineering, they showed a new method for increasing the *T*_c_ in cuprate superconductors.

H. Sato et al. [50] deposited (001)-oriented La_2−x_Sr_x_CuO_4+δ_ (x = 0 ~ 2) films by reactive electron-beam co-evaporation and characterized them by XRD and resistivity measurements. By changing the *c*-axis length, they were able to obtain single-phase films for the entire range of compositions. Films (x = 0.06 ~ 0.30) with oxygen composition *δ* ~ 4 showed superconductivity. The *T*_c_ was maximized to 44 K for x = 0.15, owing to a strain effect caused by lattice mismatch. There was a good correlation between the film’s *T*_c_ and the length of the *c*-axis. When x = 0.30, the *T*_c_ of films was strongly dependent on the residual resistivity (*ρ* (0 K)), meaning they showed a higher *T*_c_ for lower *ρ* (0 K). When x = 0.125 was used, the depression of *T*_c_ was smaller than when it was used for bulk samples.

Under a compressive epitaxial strain, I. Zaytseva et al. [51] studied the transport properties and structure evolution of underdoped La_1:952_Sr_0:048_CuO_4_ films. The films with different thicknesses (26 ~ 120 nm) were deposited. Superconductivity in films started at 26 K, but small residual resistance remained at low temperatures, suggesting a non-homogeneous state of superconductivity. There was a saturation of resistance at about 0.65 K under a perpendicular magnetic field, indicating the possible existence of a non-conventional metallic state. At a magnetic field of about 32 T, a magnetic field-tuned transition from quasi-superconductor to insulator was observed.

**Figure 4:**
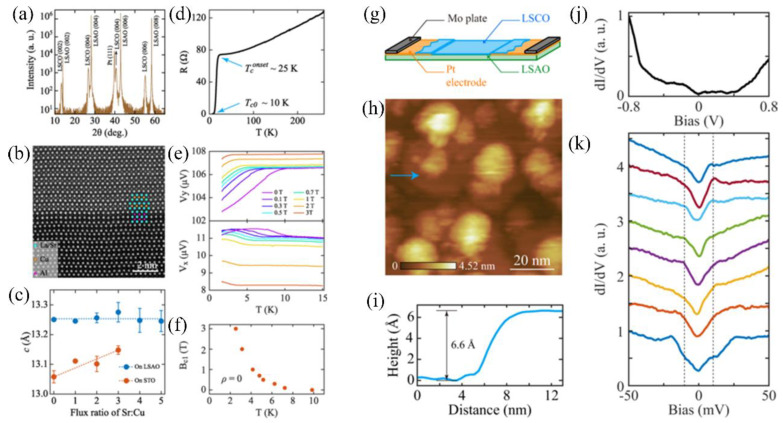
(**a**) XRD of the LSCO film. (**b**) STEM image of the LSCO/LSAO heterointerface. (**c**) Comparison of *c*-axis lattice parameters vs. flux ratios of Sr:Cu. (**d**) *R*-*T* curve of the LSCO/LSAO sample. (**e**) Imaginary and real parts of mutual inductance vs. *T* under different magnetic fields. (**f**) *B*_c1_ vs. *T* of LSCO films. (**g**) Schematic diagram of the LSCO film deposited on the Pt electrode pre-patterning LSAO substrate. (**h**) STM topography of the LSCO/LSAO sample. (**i**) Line profile along the blue arrow in (**h**). (**j**) Large- and (**k**) Low-energy-scale d*I*/d*V* spectrum in the LSCO/LSAO film. Adapted with permission from Ref. [52]. Copyright 2021, American Chemical Society.

Yang Wang et al. [52] employed molecular beam epitaxy (MBE) to deposit LSCO films. They investigated the influence of substrate-induced strains on superconductivity and crystal parameters of LSCO films through heterointerface engineering. When deposited on SrTiO_3_ substrates with 3% larger lattice constants than LSCO, the large tensile strain at the heterointerface prevented LSCO films’ superconductivity [48]. In contrast, LaSrAlO_4_ (LSAO) substrates had a smaller lattice constant than LSCO, which caused a slight compression at the heterointerface, which enhanced LSCO’s superconductivity, as shown in Figure 4.

**Figure 5:**
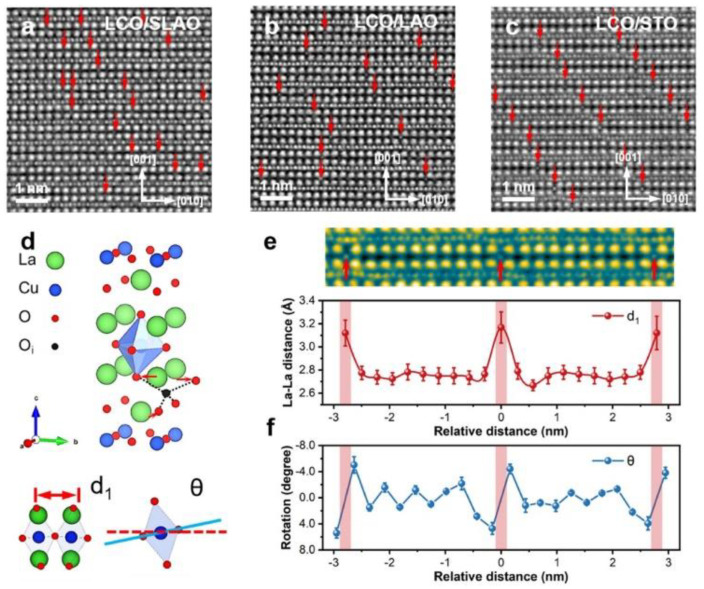
iDPC-STEM micrograph of La_2_CuO_4+δ_ deposited on (**a**) SLAO, (**b**) LAO, and (**c**) STO substrates, respectively. Red arrows illustrate the interstitial oxygen atomic columns. (**d**) Schematic structure of La_2_CuO_4+δ_. Only half of the orthorhombic cell is presented. The interstitial oxygen can lead to the slight shift of La and apical oxygen and the CuO_6_ octahedra titled. (**e**) The distance (d_1_) of La–La atomic column spacing and (**f**) the rotation of the CuO_6_ octahedra along the *b* axis vs. the relative distance from the interstitial oxygen. Reprinted with permission from Ref. [53]. Copyright 2022, Elsevier.

X. Zhang et al. [53] characterized interstitial oxygen atoms in La_2_CuO_4+δ_ by integrated differential phase contrast scanning transmission electron microscopy (iDPC-STEM) technique as shown in Figure 5. The interstitial oxygen atoms in La_2_CuO_4+δ_ lay in La_2_O_2+δ_ layers and their distribution was sensitive to local strain states and could be modulated by strain. High in-plane stress in La_2_CuO_4+δ_ created periodic oscillations in La-O layers between compressive and tensile strain. Rather than being randomly distributed, mobile interstitial oxygen atoms self-organized to form oxygen-depleted stripes which are divided by ordered oxygen interstitials. In addition, density-functional-theory (DFT) calculations revealed local changes in electronic and atomic structures. Dopant oxygen atoms could be adapted in CuO_6_ octahedrons by shifting surrounding atoms and distorting CuO_6_ octahedras. The interaction between CuO_6_ octahedras and dopant oxygen atoms induced charge transfer from LaO planes (charge carrier reservoir) to CuO_2_ planes (hole doping). By forming apical O of CuO_6_ octahedra, oxygen atoms in LaO planes created a link between O and CuO_2_ planes, increasing the hole carrier concentration of CuO_2_ planes. Their result offered a systematic view of the locations of dopant O atoms and their impact on the electronic and atomic structure of La_2_CuO_4+δ_.

### 4.2. Effect of Strain on Bi_2_Sr_2_Ca_n−1_Cu_n_O_2n+4+δ_ System

Bi_2_Sr_2_Ca_n_Cu_n−1_O_2n+4+δ_ has intrinsic strain due to its structural complexity, which is especially suitable for strain study of superconductors. The largest source of strain in Bi_2_Sr_2_CaCu_2_O_8+δ_ (Bi-2212) is the incommensurate structural buckling (a mismatch between rock-salt BiO_2_ layers and CuO_2_ planes) called “super-modulation” with a period of ~26 Å, oriented at 45° from the Cu-O bond [54,55]. The second source is a commensurate orthorhombic distortion that shifts two sublattices in opposite directions primarily in BiO planes, perpendicular to the super-modulation wavevector [56,57]. In both modulations, oxygen atoms in the lattice are distorted by more than 0.5 Å.

Bennie ten Haken et al. [58] investigated the mono and multifilamentary Bi-2212 wires in the Ag matrix by an axial strain experiment. A tensile or compressive axial strain was achieved by soldering superconducting samples to a bent substrate. In magnetic fields up to 16 T at 4.2 K, the *I*_c_-strain dependence was measured with strains ranging from −2% to +1.2%. All strain-induced reductions in *I*_c_ in Bi-2122 samples were irreversible. Moreover, the strain change did not result in a significant rise in *I*_c_. A special emphasis was placed on axial strains in the tensile regime (0 ~ 0.4%). In this case, *I*_c_ was reduced slightly but significantly. This strain behavior indicated that fractures in superconducting filaments are responsible for the *I*_c_ reduction.

The electronic inhomogeneity of HTSs may provide an opportunity to enhance superconducting pairing. Ilija Zeljkovic et al. [8] reported the correlation between local strain and regional doping in Bi-2212. Scanning tunneling microscopy (STM) revealed periodic distributions of oxygen dopants related to local strain. By investigating all oxygen dopant positions, they provided crucial structural input for a complete microscopic theory.

**Figure 6:**
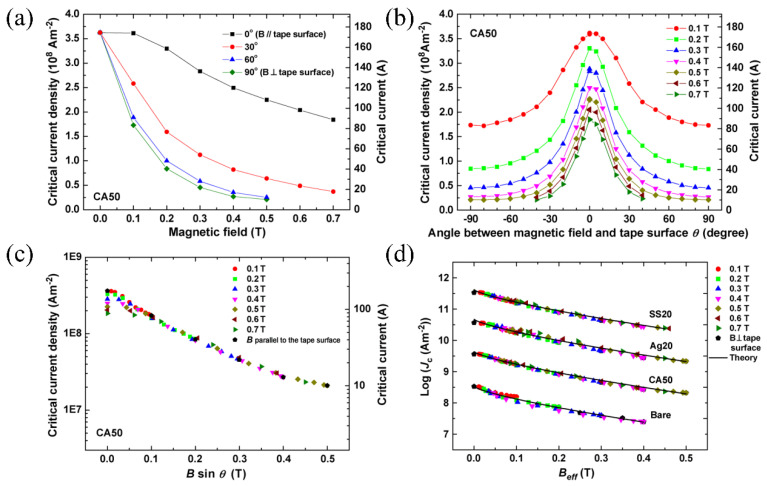
The electrical transport properties of the BSCCO tape at 77 K. (**a**) *J*_c_ vs. *B* for different field orientations. (**b**) *J*_c_ vs. ***θ*** at different applied magnetic fields. (**c**) *I*_c_ vs. *B*sin*θ* of the tape. (**d**) *J*_c_ vs. *B*_eff_ for different tapes at 77 K. Reprinted with permission from Ref. [59]. Copyright 2011, IEEE-Inst Electrical Electronics Engineers Inc.

Prapaiwan Sunwong et al. [59] investigated the impact of magnitude and direction of magnetic field on BSCCO tapes under tensile and compressive strains. The anisotropy, texture and structure of Bi_2_Sr_2_Ca_2_Cu_3_O_10+δ_ (Bi-2223) had a great influence on *J*_c_. The magnitude and angle correlations with *J*_c_ are shown in Figure 6 and could be explained by a simple anisotropic exponential magnetic field model that contained the influence of 2D and grain misalignment. In the range of reversible strain, the variation in normalized was linear, where temperature and field were not relevant to the gradient of strain dependence. Degradation by compression extended the reversibility further into the compressive regime.

L. Forro et al. [60] measured the in-plane resistivity (*ρ*_ab_) and out-of-plane resistivity (*ρ*_c_) of Bi-2212 single crystals under pressure ~20 kbar. *ρ*_ab_ had an initial derivative, *d*(ln*ρ*_ab_)/*dP* = −0.75% /kbar. However, the effects of pressure were much greater on *ρ*_c_, *d*(ln*ρ*_ab_)/*dP* = −4.0% /kbar. Throughout the pressure range covered, *T*_c_ increased monotonically by +0.15 K/kbar in all configurations.

S. Klotz et al. [61] studied the relationship between *T*_c_ and the pressure of Bi-2212 single crystals. The dependence of *T*_c_ of Bi-2212 single crystals on hydrostatic pressure to 7 GPa was measured at three different oxygen concentrations. All three cases showed markedly nonlinear *T*_c_(*P*) behavior. With increasing oxygen concentrations, d*T*_c_/d*P* = +1.2 K/GPa for the samples with *T*_c_(0) = 80 K and 88 K, followed by maximum in *T*_c_(*P*) measurements at 4 GPa and 1.5 GPa for the samples, respectively. When the oxygen-poor sample was exposed to 1.5 GPa, *T*_c_(0) did not change much. At higher pressure, *T*_c_(0) showed a downward trend, but the decrease was slow. In the absence of a pressure medium, when the anvils directly contacted the sample, *T*_c_(*P*) exhibited a markedly different pressure dependence. Models based on charge transfer cannot account for the present results.

Xiao-Jia Chen et al. [62] studied the pressure dependence of *T*_c_ up to 18 GPa of Bi-2212 single crystals with underdoped, optimally doped, and overdoped levels. For all samples, as pressure increases, *T*_c_ increased initially and saturated at critical pressure (*P*_c_) subsequently. As pressure increased, *T*_c_ decreased modestly. Oxygen doping tends to decrease the increase in *P*_c_ and *T*_c_. It was then possible to construct a high-pressure phase diagram between saturated *P*_c_ and *T*_c_. A theoretical interpretation, according to the competition between pairing interaction strength and hole carrier density, was given based on transport data of the Hall coefficient and the resistivity in this system.

**Figure 7:**
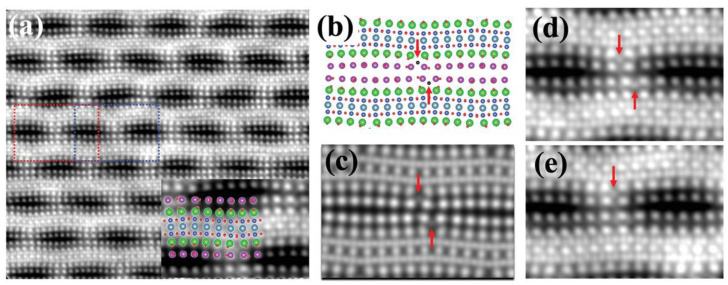
Imaging of dopant oxygen atoms. (**a**) iDPC-STEM image of Bi-2212 along *b*-axis. (**b**) Schematic diagram of Bi-2212. (**c**) Simulated iDPC-STEM image. Enlarged images taken from the dotted red (**d**) and blue (**e**) rectangles in (**a**). Reprinted with permission from Ref. [63]. Copyright 2019, Wiley.

Dongsheng Song et al. [63] observed the locations of dopant oxygen in Bi-2212 by the HAADF-STEM and iDPC-STEM techniques, as shown in Figure 7. According to iDPC and DFT results, the favorable positions of dopant oxygen between SrO and BiO can be explained by local strain analysis. Furthermore, the DFT calculations revealed local changes in electronic and atomic structure. As the surrounding atoms shift, dopant O atoms were accommodated, further aggravating the distortions. At the same time, the dopant O atoms induced charge transfer from BiO planes (charge carrier reservoir) to CuO_2_ planes (hole doping). The O atoms of SrO planes served as apical O in CuO_5_ pyramids, establishing a correlation between the dopant O and CuO_2_ planes, and improving hole carrier concentration. Their work provided an in-depth insight into the dopant oxygen atom locations and how dopant O atoms affect the electronic and atomic structure of Bi-2212.

### 4.3. Effect of Strain on the REBa_2_Cu_3_O_7−δ_ System

**Figure 8:**
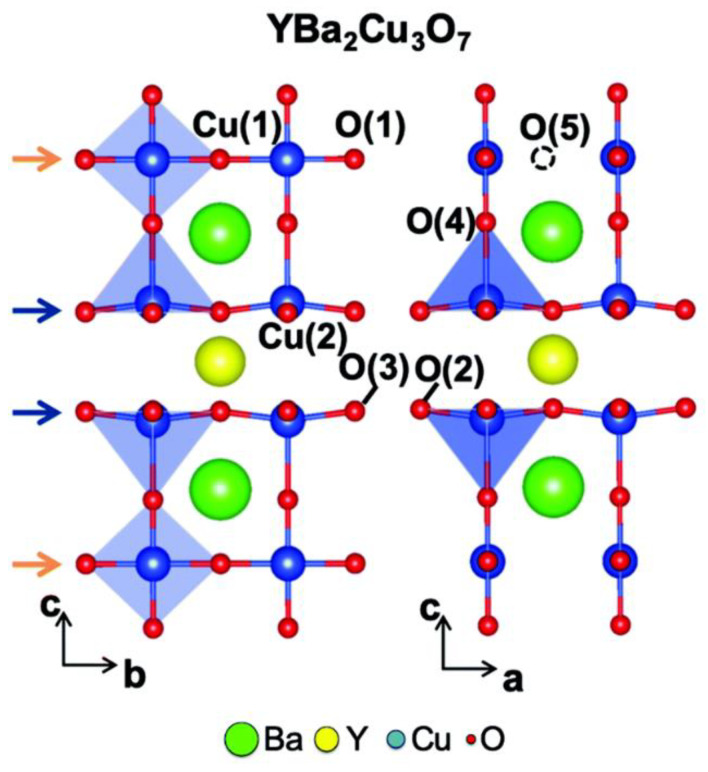
Schematic diagram of the YBCO unit cell. Reprinted with permission from Ref. [64]. Copyright 2020, Royal Society of Chemistry.

In YBCO’s unit cell structure, CuO_2_ planes are separated by oxygen-free Y layers. CuO_2_ planes are linked to Cu-O chain layers oriented along the *b*-axis by a bridging or apical O referred to as O(4) in Figure 8. Thus, orthorhombic YBCO contains five inequivalent oxygen sites.

D. C. van der Laan and J. W. Ekin et al. [65] measured the intrinsic effect of axial strain in YBCO-coated conductors. YBCO for electric power applications has been measured in a wide spectrum of strains with a new technique to demonstrate a significant reversible reduction in critical current (*I*_c_). *I*_c_ was reduced by 40% at 1% compressive strain in the self-magnetic field, and this effect was symmetric for tensile and compressive strains. A significant impact is expected from this effect and its magnitude on power applications. It can offer a new idea for exploring the basic properties of current transport in HTSs.

N. Cheggour and J. W. Ekin et al. [15] studied the impact of axial strain on *J*_c_ for YBCO coatings on different substrates. According to their study, the reversible degradation of *J*_c_ occurred at about twice the substrate’s yield strain. Axial-strain performance requirements of electric devices, such as motors and generators, requiring strain tolerances exceeding 0.25%, can be met by YBCO/Ni-alloy composites. Furthermore, the YBCO/Ni-5-at.%-W conductors exhibited a reversible strain effect, which may be induced by a reversible strain-field broadening around mismatch dislocations at grain boundaries.

Through finite element analysis, Barth et al. [66] studied the effect of twisting or torsion exerted on HTS tapes as a source of longitudinal and shear stress. Van der Laan et al. [67] reported that the strain fields surrounding grain boundary dislocations in YBCO thin films significantly suppressed the local *J*_c_. *J*_c_ increased dramatically when strain was removed from superconducting grain boundary channels by compressive strain. Grain boundary channels have strain-free *J*_c_ values comparable to intrinsic *J*_c_ values of grains.

R. Guzaman et al. [68] systematically studied the local strain distribution within YBCO films. By forming Y124, strain-derived interactions with other defects were concatenated and intrinsic defects were nucleated in YBCO films, resulting in a distributed network of randomly distributed nanostrained regions that profoundly altered vortex-pinning efficiency.

K. N. Yugay and A. V. Muravjev [69] used laser ablation to deposit strained YBCO films (thicker than the critical thickness) on LaAlO_3_(100) substrates. By rapidly cooling films after deposition, strains were frozen in films. Unusual temperature dependence of *J*_c_ was observed in these films: a characteristic minimum was observed between 55 ~ 57 K. The *J*_c_ decreased from 10^6^ A/cm^2^ to 10^4^ A/cm^2^ and was lower at 77 K. The films are stable against thermocycling from 300 K to 77 K. STM analysis showed that strain domains were formed in films, leading to a macroscopic structural organization. On average, as the strain increased, the size of domains decreased, from 2.4 to 1 μm. Magnetic fields penetrated differently into strained films than into single-crystal or granular films, which implied that the films had a macroscopic structure. 

Based on X-ray diffraction line broadening, X. Obradors et al. [70] discovered that *J*_c_ and YBCO films’ mesostrain were adversely correlated. Mesostrain was enhanced when grain boundaries were not effectively healed, for example by shortening annealing times or lowering growth temperatures. By applying the bond contraction pairing model to strained regions at low angle grain boundaries, the microscopically observed weak link behavior could be explained by pair formation prevention.

Using epitaxial YBCO films on (001)-oriented piezoelectric Pb(Mg_1/3_Nb_2/3_)_0.72_Ti_0:28_O_3_ substrates, P. Pahlke et al. [71] applied reversible and continuous biaxial strain to underdoped and optimally doped YBCO films. With an electric field applied to the substrates, the biaxial strain (~0.1%) was able to be induced into YBCO films, which resulted in a reversible and continuous shift in *T*_c_, the upper critical field, and the normal state resistance. YBCO films with optimal doping had a shift of *T*_c_ of 0.75 K per 1% compressive biaxial strain, whereas underdoped films had a shift of 4.20 K. These values matched highly with strain sensitivity data calculated from pressure experiments.

D C van der Laan et al. [21] found that YBCO-coated conductors with axial strain underwent a large, magnetic-field-dependent, reversible reduction in *J*_c_ at 75.9 K. This effect could be potentially significant in applications where YBCO coatings were subjected to high stresses and magnetic fields. Scientists constructed a device to measure *J*_c_ as a function of axial tensile and compressive strains and applied magnetic fields to determine its magnitude and origin. At all magnetic field angles, *J*_c_ reduced reversibly with strain increasing. When 8 T magnetic field was parallel to *ab*-plane of the conductor or 5 T to *c*-axis, *J*_c_ was reduced by about 30% at 0.5% strain, compared with about 13% reduction in self-field at 76 K. Different *J*_c_ strain responses at various magnetic field angles revealed that strain was uniquely affecting the pinning mechanism in YBCO-coated conductors.

D. Putzky et al. [72] utilized atomic layer-by-layer MBE (ALL-MBE) to synthesize DyBa_2_Cu_3_O_7−d_ films with minimal defect density. Orthorhombic and tetragonal structures with different twinning patterns were revealed by X-ray reciprocal-space maps. They elucidated the evolution of oxygenation state, film thickness, and epitaxial relationship with substrates. In addition, they found that films with a higher degree of orthorhombicity exhibited higher *T*_c_ and lower normal-state resistivities. Consequently, optimized superconducting heterostructures and devices can be synthesized based on these findings.

**Figure 9:**
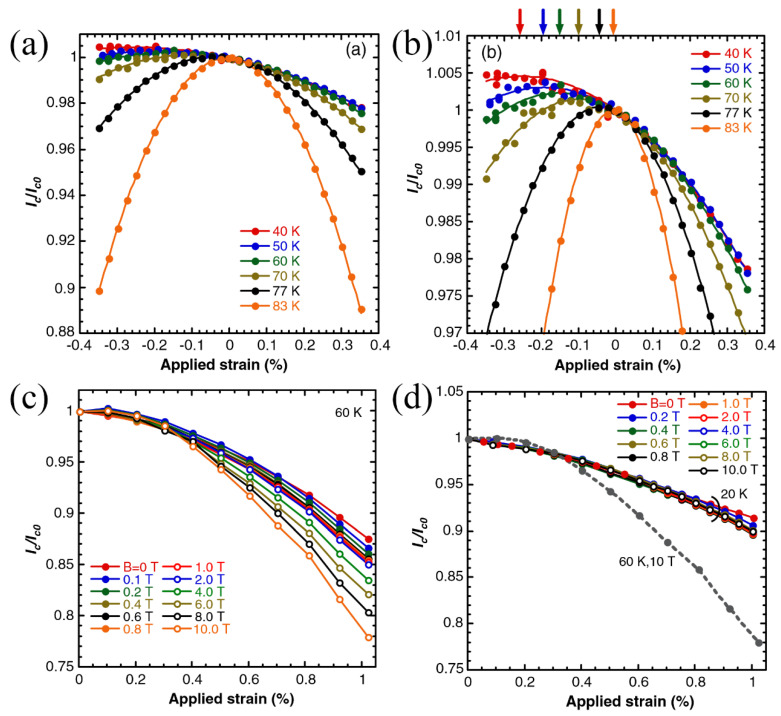
*T* vs. normalized *I*_c_ in self-field for 0.1 μm thick YBCO layers. The whole data (**a**) and corresponding enlarged view (**b**) around the peak are presented. *B* vs. normalized *I*_c_ at 60 K (**c**) and 20 K (**d**). Reprinted with permission from Ref. [13]. Copyright 2010, IOP Publishing Ltd.

The strain effect on *I*_c_ in YBCO coated conductors at 20–83 K under magnetic fields parallel to *c*-axis up to 10 T was investigated by Michinaka Sugano et al. [13], as shown in Figure 9. Across all tested temperatures, *I*_c_ exhibited reversible peak variation in self-field with applied uniaxial strain. As the temperature increased, strain sensitivity increased, which resulted in a more obvious reversible suppression with strain. In a fascinating finding, as the temperature decreased, the peak strain corresponding to *I*_c_’s maximum shifted to the compressive side. *I*_c_’s peak strain cannot be explained by changes in the residual thermal strain of YBCO films alone, as this peak shift cannot be explicated by the relaxation of residual strain. At 60 K, *I*_c_ strain sensitivity increased with the increasing magnetic field, whereas at 20 K, magnetic field influence was less pronounced. When the magnetic field was low, at 77 and 83 K, the in-field *I*_c_ displayed a double peak behavior both at compressive and tensile regions. 

It is well known that in YBCO, oxygen vacancies (V_O_s) regulate the carrier concentration, *J*_c_ and *T*_c_. Bernat Mundet et al. [64] demonstrated that V_O_ accommodated local strain fields caused by large-scale crystal defects. A significant influence on the location and concentration of V_O_ was found to be related to nanoscale strain relevant to Y_2_Ba_4_Cu_8_O_16_ (Y248) intergrowths. Based on DFT calculations and local probe measurements, it appears that strain caused the V_O_ to reorder from CuO chains into the bridging apical sites in BaO planes, where it bound directly to superconducting CuO_2_ planes. Their results have strong connotations on the physical properties of YBCO, because apical V_O_s altered carriers transfer to CuO_2_ planes, which is identified by electron energy loss spectroscopy (EELS), and created structural changes. Further, the discovery of apical V_O_s also had an influence on regulating *J*_c_ and enhancing the vortex pinning process.

### 4.4. Effect of Strain on Other Superconductor Systems

Alongside cuprate superconductors, lattice strain is also important in other types of superconductors. For example, in FeSe system, *T*_c_ was enhanced by growing epitaxial thin films on SrTiO_3_ substrates (from 8 K for bulk material to above 65 K) [73,74,75]. Both electronic structure reconstruction and biaxial lattice strain seemed to have an influence on improving *T*_c_ by charge transfer from oxygen-vacancy-induced states of substrates.

S. J. Zhang et al. [76] measured the resistance of Li_x_FeAs (x = 0.8, 1.0, 1.1) superconductors under high pressure. It is found that *T*_c_ decreased almost linearly with the increase in pressure and pressure derivative (*dT*_c_/*dP*). In situ synchrotron radiation powder, X-ray diffraction experiments at high pressure demonstrated no correlation between structural transitions and resistivity.

Under uniaxial compressive stress, Ekin [77] observed a large reversible degradation of *I*_c_ in Nb_3_Sn [77]. In addition, Ekin [77,78] also studied the *H*_C1_ under transverse compression stresses. *H*_C1_ of Nb_3_Sn was intrinsically affected by transverse stress ten times more than by axial stress. Further investigations of the strain’s influence on *H*_C1_ have been published in recent papers [79,80,81].

A.V. Pogrebnyakov and J. M. Redwing [82] reported that tensile strain led to a remarkable increase in *T*_c_ in MgB_2_ films, which was well beyond the bulk value. Due to the coalescence of initially nucleated discrete islands, tensile strain increased with increasing MgB_2_ film thickness. On sapphire and SiC substrates, epitaxial films showed *T*_c_ increases, but the values for the two types of substrates differed due to lattice mismatch and thermal expansion mismatch. According to first-principles calculations, the increase in *T*_c_ is due to a softening of the bond-stretching E_2g_ phonon mode, which is defined by Raman scattering. According to their results, *E*_2g_ phonon softening was an option for achieving higher *T*_c_ with MgB_2_-related materials.

Y. C. Liu et al. [83] investigated the superconductivity of Sr_2_RuO_4_ films under strain. Their experiments showed that tensile strain caused charge transfer to the γ-band and consequent Lifshitz reconstruction of the Fermi surface. According to theoretical calculations, a *p*-wave superconducting order was realized with enhanced *T*_c_ within a limited range of tensile strain. With an increase in strain, however, before reaching the Lifshitz transition, the system had developed into the spin-density wave (SDW) state. They also considered the effect of compressive strains. A transition from the *p*-wave SC state to the nodal *s*-wave SC state occurs as strain increases. A comparison of theoretical results to the experiment can be made, and further experiments can be performed to verify the results. As biaxial strain played a vital role in modulating the system to Van Hove filling, they predicted that *T*_c_ may also rise rapidly on a fixed substrate but the thin film is slightly gated toward higher filling.

It has been several decades since SrTiO_3_, the first oxide superconductor was discovered, but its nature of superconductivity remains a source of intense debate. Recent theoretical studies have implied the incipient ferroelectric properties and superconductivity of SrTiO_3_ may be linked. K. Ahadi [84] studied the phenomenon of enhanced superconductivity of SrTiO_3_ films under strain state. They grew strained, epitaxial SrTiO_3_ films, which underwent ferroelectric transitions, to test whether such a connection exists. Within a range of carrier densities, when compared with unstrained films deposited under the same conditions, *T*_c_ was enhanced by up to a factor of two. Additionally, superconductivity emerged from a resistive state in these films. Localization behavior was discussed in the context of ferroelectricity proximity. The results provided new chances to enhance *T*_c_ in oxide materials.

Through calculation, Mano et al. [85] found that Li-doped MoS_2_ thin films performed differently in tensile and compression states, which was believed to result from the change in topology structure of the Brillouin region on the Fermi surface and the increase in electron-phonon matrix elements. Using ab initio calculations, they showed that tensile or compressive strain could enhance the superconductivity of Li-intercalated bilayer MoS_2_. It was found that the mechanism of superconductivity enhancement differed for tensile strains and compressive strains. In a large range of Brillouin zones, it was possible to explain enhanced electron-phonon coupling (EPC) under tensile strain as a result of a change in Fermi surface topology, increasing the nesting function. When 6.0% tensile strain was applied, the *T*_c_ of 0.46 K at zero strain increased to 9.12 K. Moreover, increased intrinsic electron-phonon matrix elements can explain the enhancement under compressive strain. Additionally, electron pockets on Fermi surfaces played a significant role in interband scattering. Finally, approximately 80% of the overall EPC (λ = 0.98) originated from these pockets and the *T*_c_ was estimated at 13.50 K.

### 4.5. Effect of Strain on Secondary Phase Vortex Pinning

In high-*T*_c_ cuprate superconductors, lattice distortion can modify pairing, resulting in enhanced vortex pinning due to nanostrain. Hence, a comprehensive insight into the strain landscape is necessary for large-scale HTS applications. However, identifying defects’ type and distribution, along with associated strain, is crucial work.

**Figure 10:**
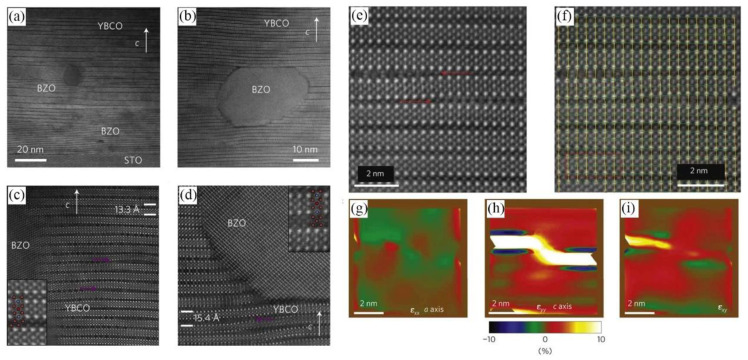
Z-contrast images of BZO nanoparticles in YBCO matrix at low (**a**) and high (**b**) magnification. (**c**,**d**) High-resolution Z-contrast images present the region between two different BZO nanoparticles. The insets in (**c**,**d**) show intergrowth in detail. (**e**) Z-contrast image where the strain maps were generated. (**f**) Grid obtained by Peak Pairs Analysis (PPA) from (**e**). (**g**–**i**) ɛ_xx_, ɛ_yy_ and ɛ_xy_ maps, respectively. Reprinted with permission from Ref. [86]. Copyright 2012, Nature Portfolio.

A. Llordés et al. [86] demonstrated a highly effective artificial pinning centers (APCs) mechanism in HTS nanocomposite films by generating nanostrained regions which suppressed the formation of Cooper pair formation. Nanostrained regions characterized by TEM revealed highly concentrated dislocations relevant to intergrowths between epitaxial YBCO matrix and randomly oriented APCs, as shown in Figure 10a–d. Randomly oriented oxide nanoparticles caused nanoscale defects to occur in YBCO matrix, especially YBa_2_Cu_4_O_8_ (Y248) intergrowths (extra Cu-O chains), which eventually resulted in highly localized strained areas where Cooper pair formation was suppressed [86,87]. Consequently, the nanostrain significantly enhanced vortex pinning in randomly oriented APCs. By quenching Copper pair formation under tensile strain, a new and effective vortex pinning mechanism was proposed based on the bond contraction pairing model, as shown in Figure 10e–i.

Pablo Cayado et al. [88] utilized the CSD method to deposit GdBa_2_Cu_3_O_7−d_ (GdBCO) and GdBCO–Gd_2_O_3_ nanocomposite films. The structural and physical properties of GdBCO films were remarkable, presenting an (*00l*) epitaxial orientation, with *T*_c_ values achieving 92.8 K, which was an increase of more than 1 K over standard YBCO films. As a result of the presence of Gd_2_O_3_ nanoparticles, nanostrain values reached 0.24 ± 0.01% in GdBCO–Gd_2_O_3_ nanocomposite films, higher than those of GdBCO-low fluorine films (0.13 ± 0.01%). Aside from that, the nanostrain obtained in GdBCO–Gd_2_O_3_ nanocomposite films was comparable with that found in CSD YBCO nanocomposites.

Roger Guzman et al. [68] applied scanning transmission electron microscopy (STEM) to analyze the microstructure of individual defects in YBCO nanocomposite films. Due to the introduction of incoherent secondary phase nanoparticles, the density of Y_1_Ba_2_Cu_4_O_8_ (Y124) intergrowths increased significantly. It has been shown that the formation of Y124 triggered strain-induced interactions with other defects as well as intrinsic defect nucleation. These interactions resulted in a net of randomly distributed nanostrained regions, transforming the vortex pinning landscape profoundly. It is believed that the additional structural strain, triggered by nanoparticles and corresponding induced defects, enhanced the vortex pinning [89].

It is well known that Ba*M*O_3_ (B*M*O, *M* = Zr, Hf, Sn, etc.) self-organizes into nanocolumns in *RE*BCO films deposited by vapor phase epitaxy. C. Cantoni et al. [90] reported that oxygen defects were induced at the contact surface between BaZrO_3_ and YBCO matrix due to strain, leading to a strong vortex pinning effect, and thus, significantly increasing *J*_c_ of YBCO superconductors. A higher concentration of BaZrO_3_ nanocolumns results in a larger entire tensile strain of the *RE*BCO along the *c*-axis, causing an average increase in *c*-axis lattice parameter.

Jiachao Ye et al. [91] prepared 1 mol% and 3 mol% of BaHfO_3_-doped Y_0.5_Gd_0.5_Ba_2_Cu_3_O_7−δ_ (YGdBCO) nanocomposite films with a micron level thickness on CeO_2_ layers under different laser fluences respectively by PLD. Under a higher laser fluence, compared with 1 mol% films, 3 mol% of BaHfO_3_-doped YGdBCO films had lower self-field *J*_c_ at 77 K, dropping from 2.143 to 1.75 MA/cm^2^, even though there was a smoother surface. When the laser fluence was lower, both films had poorer surface morphology and superconductivity, which could be explained by the appearance of *a*-axis-oriented grains and disordered nanoparticles on the surface attributed to reduced diffusion energy. Moreover, the superconductivity decreased with increasing BaHfO_3_ doping concentrations. As a result of lattice mismatch, strain fields would further reduce diffusion energies of YGdBCO atoms, making it easier to form *a*-axis-oriented grains and degradation of surface quality, thereby aggravating the reduction in *I*_c_.

S Miura et al. [92] fabricated BaHfO_3_-doped SmBa_2_Cu_3_O_y_ (SmBCO) nanocomposite films via PLD with the low-temperature growth (LTG) technique. With the LTG technique, high-quality SmBCO films can be fabricated at low substrate temperatures during deposition, and BaHfO_3_ nanocolumn density can be greatly increased. By TEM observations, they identified that 5.6 vol% BaHfO_3_-SmBCO films had a high density [(4.8 ± 0.3) × 10^3^ μm^−2^] of pony-size (5.4 ± 0.7 nm) BaHfO_3_ nanocolumns. In 9 T, maximum flux pinning force (*F*_p_) was 405 GN m^−3^ for 40 K and 105 GN m^−3^ for 65 K. It is believed that the small size and high density of nanocolumns in SmBCO matrix can enhance *F*_p_ at low temperatures under a high magnetic field. Subsequently, Yusuke Ichino et al. [93] deposited SmBCO films with 16 vol% BaHfO_3_ using PLD and LTG techniques. As compared with 5.6 vol% BaHfO_3_-doped samples, BaHfO_3_ nanocolumns had a higher number density and a smaller diameter. Due to the substantial BaHfO_3_ content, conventional X-ray diffraction could readily evaluate the lattice strain exerted on SmBCO and BaHfO_3_. Based on this evaluation, the tensile strain on SmBCO decreased with decreasing substrate temperature, whereas the compressive strain on BaHfO_3_ increased. Results suggested that *RE*BCO was not significantly influenced by narrow nanocolumns exerting stress. Due to the small amount of stress induced by B*M*O nanocolumns, LTG proved to be an efficient method for preparing *RE*BCO films with high-density B*M*O nanocolumns.

S V Samoilenkov et al. [94] performed accurate X-ray diffraction studies on BaZrO_3_, BaCeO_3_ and Y_2_O_3_ doped epitaxial YBCO nanocomposite films. It was found that all of the introduced secondary phases were expanded within the *ab*-plane and compressed along the *c*-axis. The tetragonal distortion increase was observed in the row BaZrO_3_-BaCeO_3_-Y_2_O_3_. In the post-deposition oxygenation step, the *c*-axis lattice parameter of YBCO decreased abnormally large, resulting in anisotropic strains. This suggestion was supported by experimental observations on reoxygenated and reduced samples. As the oxygen content of YBCO changes, nanoinclusions’ strain appeared to be reversible.

**Figure 11:**
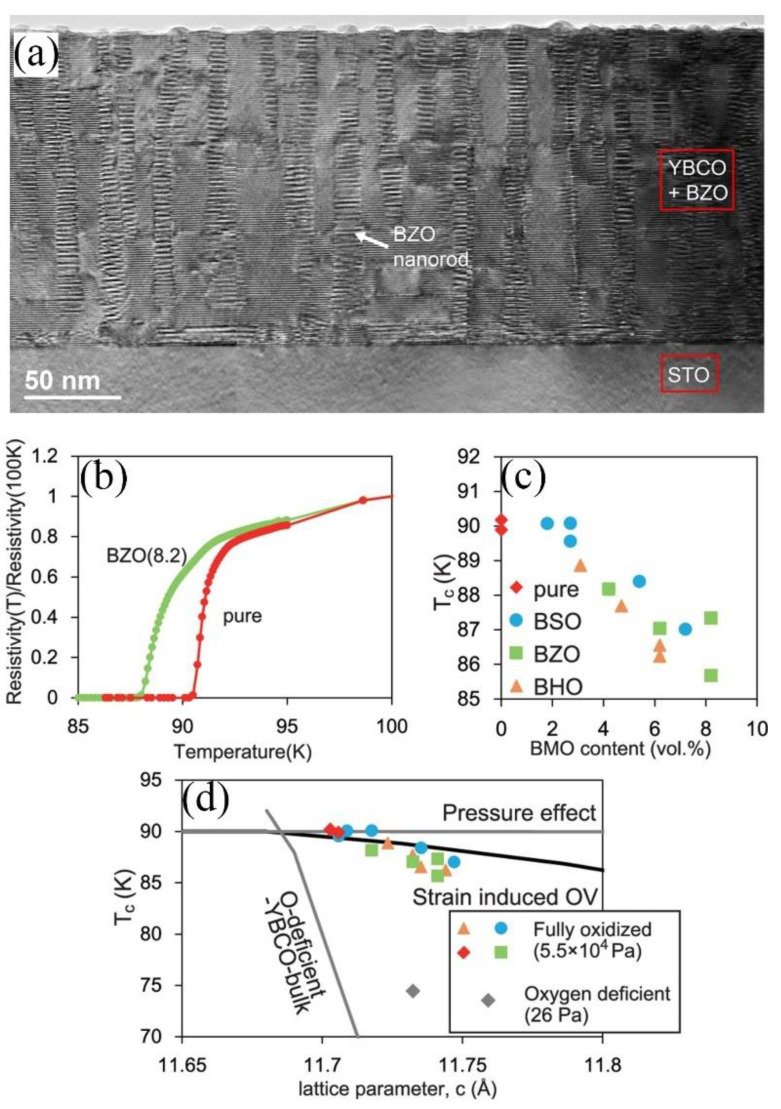
(**a**) BF-STEM image of the YBCO+BZO film. (**b**) *T* vs. normalized *R* in the pure YBCO and YBCO+BZO films. (**c**) *T*_c_ vs. B*M*O content in nanocomposite films. (**d**) *T*_c_ vs. *c*-lattice parameters. Reprinted with permission from Ref. [95]. Copyright 2017, American Chemical Society.

Tomoya Horide et al. [95] fabricated BaZrO_3_, BaSnO_3_, and BaHfO_3_-doped YBCO nanocomposite films on SrTiO_3_(100) substrates by PLD. Their work characterized and modeled the highly elastically strained nanocolumns, the heterointerface region with dislocations and distortion, as well as the matrix with oxygen vacancies induced by strain, as shown in Figure 11a. Large misfit strains were elastically accommodated by nanocolumns so the concentration of oxygen vacancies was small enough for YBCO to maintain high *T*_c_ (>85 K). The microstructure of YBCO was distorted by the interfacial bonding distorted; however, the distortion thickness was limited to several unit cells (less than coherence length) because of the electron screening. In nanocomposites, the influence of volume fraction on electron screening and the elastic strain was critical for strong vortex pinning without significant degradation of *F*_p_ and *T*_c_, as shown in Figure 11b–d. 

Masaya Gondo et al. [96] deposited double perovskite Ba_2_LuNbO_6_(BLNO) doped YBCO films on SrTiO_3_ substrates by PLD, and corresponding nanostructures were characterized by TEM and STEM. By cross-sectional observations and elemental mapping, it has been demonstrated that BLNO was self-assembled, resulting in nanocolumns stretching straight from the substrate to the surface of YBCO films. The formation of BLNO nanocolumns was disturbed by stacking faults perpendicular to the growth direction. GPA strain maps showed that tensile strain occurred around BLNO nanorods in the YBCO matrix. At the heterointerface between the nanocolumns and matrix, misfit dislocations were periodically introduced, resulting in inhomogeneous strains of YBCO around BLNO nanocolumns. To determine their superconducting properties, YBCO nanocomposite films with normal and double perovskite nanocolumns were compared.

Cantoni et al. [90] showed that the oxygen composition of self-assembled superconducting films containing BaZrO_3_ nanocolumns was strongly affected by nanoscale strain modulation. By increasing the concentration of BaZrO_3_ nanocolumns, the *RE*BCO underwent a greater overall tensile strain along *c*-axis, leading to an increase in *c*-lattice parameter. Their finding explained the observed reduction in *T*_c_. Nanostrain and corresponding effects on anion composition had been demonstrated directly. Within a few nanometers of BaZrO_3_ nanocolumns, the strain was localized. 

### 4.6. Use of Buffer Layers to Release Strain

**Figure 12:**
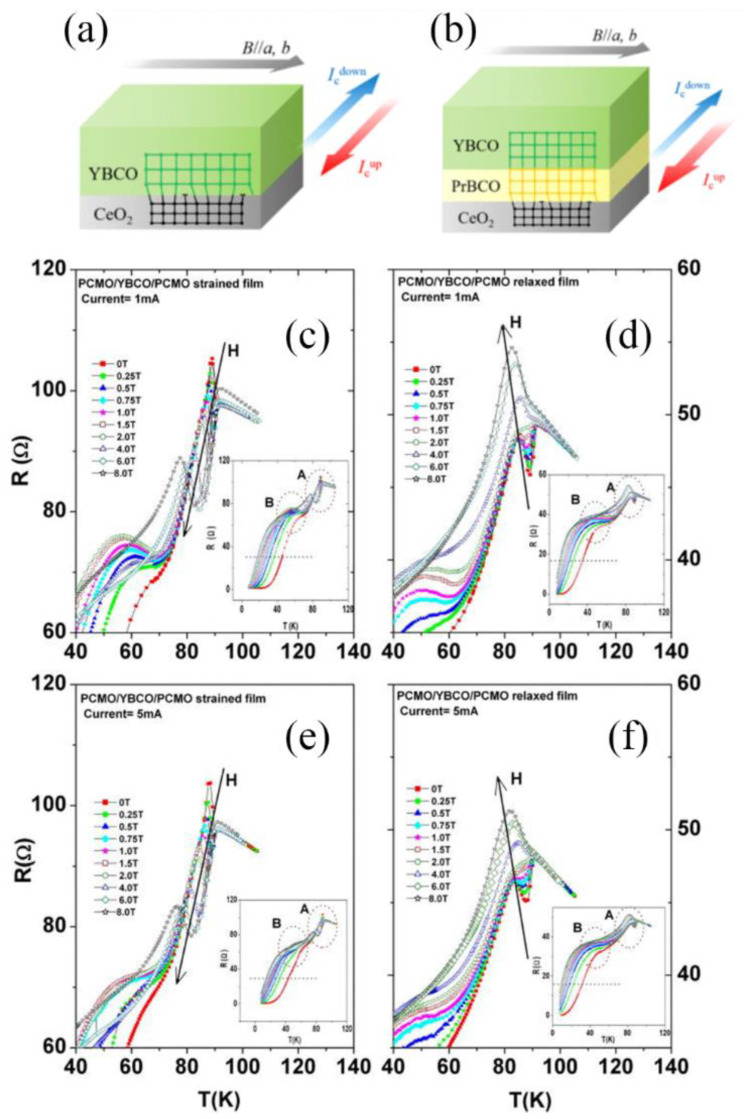
Schematic diagram of (**a**) YBCO and (**b**) YBCO/PrBCO films. Reprinted with permission from Ref. [97]. Copyright 2022, IEEE-Inst Electrical Electronics Engineers Inc. *T*-*R* behavior near the hump region at different applied *H* (0–8 T) for strained films (**c**) and relaxed films (**d**) at 1 mA applied current. (**e**) Strained film and (**f**) relaxed film at 5 mA applied current. Reprinted with permission from Ref. [98]. Copyright 2013, AIP Publishing.

By modulating the lattice strain at the heterointerface of *RE*BCO films, Akihito Mizuno et al. [97] aimed to realize superconducting diodes with rectifying properties. They fabricated YBCO/PrBa_2_Cu_3_O_y_ (PrBCO) films to improve rectifying properties, where PrBCO was used to reduce the strain resulting from the lattice mismatch between YBCO films and CeO_2_ substrates. Moreover, they assessed the rectification characteristics at different magnetic fields and temperatures to determine optimal operating conditions. Accordingly, they confirmed that maximum rectification was achieved at 0.096 T and 70 K, as shown in Figure 12.

Dipak Kumar Baisnab et al. [98] deposited trilayer films of Pr_0.5_Ca_0.5_MnO_3_/YBCO/Pr_0.5_Ca_0.5_MnO_3_ on MgO substrates by PLD. In one trilayer, the bottom Pr_0.5_Ca_0.5_MnO_3_ layer was thin, and thus, subjected to strain induced by strain. However, in another trilayer, the bottom layer was thick enough to prevent such strain from occurring. To explore how charge-order melting and the formation of ferromagnetic clusters in Pr_0.5_Ca_0.5_MnO_3_ films influence the superconductivity of YBCO, current and magnetic field-dependent resistance measurements were carried out on the trilayers at liquid helium temperatures. Compared with other trilayers, the suppression of strain on *T*_c_ of YBCO was relatively high. Based on the above data, the activation energy of vortex hopping in the sandwiched YBCO film was evaluated. The strained trilayer showed higher vortex pinning forces. Using strain, current and magnetic field simultaneously was found to be possible for controlling the superconducting behavior of sandwiched YBCO layers.

By DC magnetron sputtering, Yilun He et al. [99] grew epitaxial electron-doped infinite-layer Sr_0.9_La_0.1_CuO_2_ (SLCO) films. As well as this, they investigated corresponding structural and electrical properties by varying the film thickness and reduction annealing time. The epitaxial buffer layers of Ba_y_Sr_1−y_TiO_3_ with y = 0.4, 0.55, and 0.7, deposited on (001) (La_0.18_Sr_0.82_)(Al_0.59_Ta_0.41_)O_3_ (LSAT) substrates, were applied to introduce different types of strain to SLCO films. Short time reduction annealing of tensile strained SLCO films on Ba_0.55_Sr_0.45_TiO_3_ and Ba_0.7_Sr_0.3_TiO_3_ layers was proved to be effective for promoting superconductivity. However, reduction annealing was not effective for compressive strained SLCO films on Ba_0.4_Sr_0.6_TiO_3_ layers. Additionally, superconducting properties of tensile strained SLCO films on Ba_0.55_Sr_0.45_TiO_3_ and Ba_0.7_S_r0.3_TiO_3_ layers were strongly dependent on thickness.

C. Park and D. P. Norton et al. [100] deposited superconducting YBCO/CeO_2_/YSZ/CeO_2_ multilayer on rolled-textured (001)Ni by PLD. YBCO films with relatively thick thickness achieved *J*_c_ greater than 1 MA/cm^2^. Furthermore, *I*_c_ of YBCO films grown on rolling-assisted biaxially textured substrates was measured for their tensile and compressive bend strain tolerances. When applied compressive bend diameters up to 1.5 cm and tensile bend diameters up to 3.2 cm, these conductors retained up to 80% of their unstrained *I*_c_. *J*_c_ degraded as a result of transverse cracks forming and propagating. These results suggested an association between oxide layer thickness and bend-strain tolerance.

## 5. Conclusions and Future Outlook

The impact of strain on superconductivity is significant. The interactions between the superconductivity with deformations of the crystal lattice have already been fulfilled in experiments. Condensed matter research has been driven by the desire to increase *T*_c_ by structural and chemical manipulation for many years.

At present, the corresponding theoretical research work has also made some progress. In general, HTSs are electronically inhomogeneous at the nanoscale. It is astonishing to find that cuprate superconductors exhibit a wide range of nanoscale inhomogeneities in vital properties, such as collective mode energy (Ω) [101], the spectral gap (Δ) [102,103], and even the pairing temperature (*T*_p_) [104]. It has been debated for more than two decades which local variable is responsible for this electronic inhomogeneity. Among them, both strain [105] and dopants [106] have been empirically linked to electronic structure. Charge [107] or strain [108], or a carefully tuned combination of both [109], has been argued to be the dominant factor in theoretical models. The apical oxygen height has been closely linked to the superconducting pairing strength. Different strain theories predicted increased [17,18,110] or reduced [19] pairing strength with increasing apical oxygen height. Nevertheless, microscopic theories of reason and influence have been stalled by uncertainty about the location of dopants.

The large-scale application of superconductors in the fields of energy, information, transportation, space exploration, etc., will generate huge economic benefits. At the current stage, the application of superconductivity has not only stayed in the conceptual stage, but has been fully proven of feasibility. The emergence of new driving forces, such as performance, cost-effectiveness, reliability, practicality and competitiveness of existing technologies are the research priorities for future large-scale superconducting practical application technologies.

In this review, we summarize the current progress of strain studies on cuprate superconductors. In summary, current strain studies on superconductors are still focused on the post-preparation performance testing and analysis stages. At the preparation stage, strain magnitude cannot be controlled systematically. Although some papers have mentioned that the parameters and film thickness at the preparation stage determine the strain magnitude of films, there is still a lack of systematic guidelines and methods on how to precisely regulate the strain magnitude of the film. In addition, since the current theoretical research on cuprate superconductors lags behind experimental research, the mechanism of the effect of strain on properties is still controversial, which also limits the further development of research on HTS materials. Strain is a factor that can significantly affect superconducting properties. How to achieve the low-cost, high-reliability application of stable and controllable strain is the next urgent goal. This needs to be explored in depth before the large-scale application of HTS films.

## Figures and Tables

**Table 1:** Comparison of TEM strain measurement techniques.

Technique	Precision	Spatial Resolution	Field of View	Advantages	Disadvantages
HR-(S)TEM	10^−3^	1 ~ 2 nm	150 × 150 nm	High availability	Demanding specimen preparation and limited in field of view
NBED	10^−3^	5 ~ 10 nm	—	Practical and versatile	Low spatial resolution
CBED	2 × 10^−4^	0.5 ~ 2 nm	—	Most accurate technique	Easily interfered by bending atomic columns
DFEH	2 × 10^−4^	2 ~ 4 nm	1500 × 500 nm	Largest view areas	Low spatial resolution

## Data Availability

Not applicate.
